# Precise hepatectomy in the intelligent digital era

**DOI:** 10.7150/ijbs.39387

**Published:** 2020-01-01

**Authors:** Hao Chen, Yuchen He, Weidong Jia

**Affiliations:** 1 Department of Hepatic Surgery, The First Affiliated Hospital of USTC, Division of Life Sciences and Medicine, University of Science and Technology of China, HeFei, 230001, China; 2 Anhui Province Key Laboratory of Hepatopancreatobiliary Surgery, HeFei, 230001, China; 3 Xiangya School of Medicine, Central South University, ChangSha, 410008, China

**Keywords:** liver surgery, digital medicine, big data, artificial intelligence

## Abstract

In the past 20 years, the concept of surgery has undergone profound changes. Surgical practice has shifted from emphasizing the complete elimination of lesions to achieving optimal rehabilitation in patients. Collaborative optimization of surgery consists of three core elements, removal of lesions, organ protection and injury close monitoring, and controlled surgical intervention. As a result, the traditional surgical paradigm has quietly transformed into a modern precision surgical paradigm. In this review, we summarized the latest breakthroughs and applications of precision medicine in liver surgery. In addition, we also outlined the progresses that have been made in precision liver surgery, the opportunities and challenges that may encountered in the future.

## Introduction

Since Langenbuch implemented the world's first successful liver resection in 1888, liver surgery has developed tremendously 1. Over the past century, with the advancement of keratectomy, the safety and surgical outcomes of liver resection have been significantly improved. The perioperative mortality of liver resection has dropped from 20% in the 1970s to less than 3% in the 1990s. Some even reported zero death in more than 1000 cases of massive liver resection. The 5-year survival rate of the iconic liver cancer resection has increased from approximately 16% in the 1970s to 40% to 50% in the 1990s 2-4. Since then, liver surgical procedures have been well-established and have led to the gradual applications in a variety of diseases. Liver resection is required for patients with liver cancer, intrahepatic bile duct stones, liver trauma, and liver abscess. There is an increasing number of patients present for liver surgery, demand for better care, and concern about poor outcomes. Therefore, solutions to improve diagnosis and outcomes while driving down the healthcare cost is currently the most discussed topic in medical field.

Precision medicine (PM) is a novel medical model that was first proposed in 2011 by the National Research Council of the United States in a book named “Towards Precision Medicine: Building a Knowledge Network for New Taxonomy in Biomedicine and Disease”^5^. PM proposes customization in healthcare through medical decisions, treatments, practices, or products being tailored to individual patient. In this model, diagnostic testing is often employed for selecting appropriate and optimal therapies based on the context of the patient's genetic content 6 and other molecular or cellular analysis. Tools employed in PM include molecular diagnostics, imaging, and analytics.

In 2006, Professor Dong first proposed the concept of “precision liver surgery” to the medical world 7, 8. As a newly introduced surgical concept and technical system, precision liver surgery focuses on providing thorough preoperative evaluation, validated surgery plan, detailed surgical procedures, and excellent postoperative management at a reduced cost with minimal risk. The concept of precision liver surgery plays an important role in promoting the finest clinical practice in hepatobiliary surgery to meet the increasing demand for healthcare in this contemporary society. High demand for healthcare has given rise to technological revolution in the field of hepatobiliary surgery at a global scale. As a consequence of rising technological revolution, the therapeutic effect of hepatobiliary surgery has been improved.

As digital age is approaching, hepatectomy is undergoing a third life science revolution. Advancements in medical technology have contributed to the development of new techniques represented by artificial intelligence such as imaging omics, three-dimensional (3D) visualization and printing, molecular fluorescence imaging, multi-modal image real-time surgical navigation, etc. The trend of applying digital intelligent for diagnosis and treatment is approaching. This review intends to introduce the techniques using intelligent digital in hepatectomy from the following aspects: 1. Medical big data and artificial intelligence in the diagnosis of liver tumors; 2. 3D visualization, virtual simulation surgery and 3D printing technology to guide preoperative evaluation and surgical mode selection; 3. Molecular fluorescence imaging technology and virtual reality technology to navigate during surgery to achieve precise resection of the lesion; 4. Predictive models to guide post-operative management and the application of wearable devices as accelerated approaches to post-operative rehabilitation.

## Medical big data and artificial intelligence in the diagnosis of liver tumors

### Medical big data optimize the diagnosis and treatment strategy for liver tumors

With the aid of medical science and technology, medical data has been growing at an unprecedented rate since the late 2000s. On January 28, 2016, former President Obama signed a presidential memorandum to launch the White House Cancer Moonshot, a program to increase awareness of the importance in accelerating progress towards cancer prevention and treatment. The program emphasizes the importance of breaking down the data barriers to create a comprehensive and effective global cancer knowledge network that can accelerate the integration of genomics, epidemiology and clinical information to enable precise treatment of cancer. The completion of the Human Genome Project and the production of massive molecular biology data such as proteomics, transcriptomes, and metabolomes are the cornerstones of precision medicine 9, 10. The development of big data analysis methods and advanced detection technologies such as second-generation sequencing technology are the driving force for the development of Proteomics precision medicine. Digital gene expression profiling (DGE) uses advanced high-throughput sequencing and high-performance computational analysis techniques to detect the expression of a single gene at a specific time point in a comprehensive, rapid, and economical manner. The DGE result is represented by digitization, allowing us to have a more precise and accurate result 11-13.

DGE is currently applied to the basic hepatic carcinoma molecular research. Although the application of DGE has been making progressions in the research field, it has low clinical conversion rate. To overcome this obstacle, we should combine these high-throughput genomics, proteomics, and metabolomics analyses with high-quality and completed clinical information. In part to increase the number of potential therapeutic targets, investigators from The Cancer Genome Atlas (TCGA) Research Network identified genomic alterations in 196 tumors that influence development of HCC, including mutations in the TERT gene promotor, mutations in the TP53 and CTNNB1 (β-catenin) genes, and elevated expression of several immune checkpoint genes 14. Integrated analytic approaches have been applied to multiple data platforms from a large set of clinically annotated HCC to provide a better understanding of molecular targets that may lead to improved therapeutic strategies.

In April 2018, China National Liver Cancer Center launched a multi-center, prospective liver cancer screening program named PreCar. The project is planned to establish a follow-up cohort study, with 1-1.5 million high-risk populations of liver cancer and conduct a three-year prospective cohort study. Early detection serum markers for liver cancer will be screened, identified and applied into clinical use by using mature high-throughput genome sequencing techniques. Optimizing early liver cancer treatment strategies through big data results and provides high-quality evidence-based medicine, spares patients from suffering complex and expensive care later. While exploring the molecular mechanisms and driving genes of liver tumors, all results will be shared to the clinical. With the guidance of gene mutations biomarkers, we can apply the most appropriate treatments such as chemotherapy, radiotherapy, targeted therapy, and immunotherapy to individual patients.

The advent of the era of big data has enabled a large amount of untapped medical materials to be available for quantitative analysis such as surgical video. At present, a large number of surgical videos can be quantitatively analyzed through machine learning, using computer vision principles to achieve automatic, real-time analysis and segmentation of surgical procedures. Sharing these videos with other surgeons can also help to improve their surgical skills or for educational purposes 15-18.

### Artificial intelligence assisted diagnosis and treatment of liver tumors

Artificial intelligence is a new technology that develops theories, methodologies, and applications for simulating and extending human intelligence. The application of artificial intelligence in medical field allows computer algorithms to recognize patterns, approximate conclusions, and suggest treatments 19, 20. In 2016, the artificial intelligence robot AlphaGo, developed by Google's DeepMind, defeated the master of the Go game, marked the rising of artificial intelligence 21. The Watson for Oncology artificial intelligence-assisted diagnosis and treatment system introduced by IBM (International Business Machines) is a successful application of artificial intelligence in the medical field 22. This system can provide intelligent diagnosis and treatment plans according to the level of evidence-based medical evidence for physicians to choose based on the symptoms and examination data of patients with liver cancer.

Machine learning, a system that can learn, identify and make decisions from data, is a current application that supports artificial intelligence. Deep learning is a subset of machine learning, it is capable to establish and imitate the neural network of human brain for analysis and learning. The mechanism of imitating human brain learns the data automatically features in multi-layer structure and improves the accuracy of classification and prediction. Accurate diagnosis is an important factor in the implementation of precision medicine. Artificial intelligence-based Radiomics research is currently the most discussed research topic. With the development of image recognition technology, artificial intelligence technology can extract the subtle texture features in images, predict the pathological features of microvascular invasion, neurological invasion, lymph node metastasis and other prognosis before surgery, and guide individualized treatment. Furthermore, artificial intelligence technology can help to identify benign and malignant liver tumors, diagnose rare pathological types of liver tumors, and guide clinical diagnosis and treatment 23, 24.

**Figure 1:**
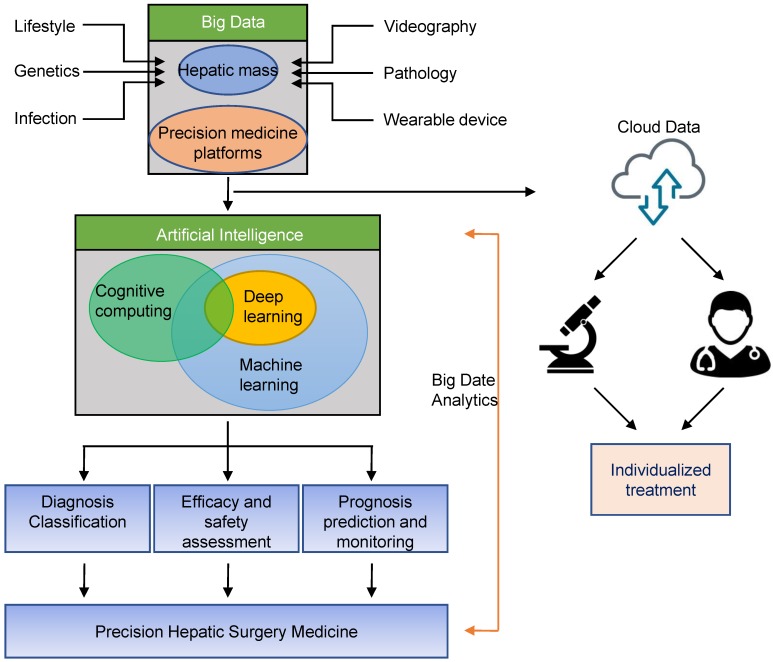
** Schematic diagram of application of Artificial Intelligence in precision hepatic surgery medicine.** Big data can be stored through liver tumor or precision medicine platforms, and can be shared for data analysis with other physicians or researchers through secure cloud systems. Big data analytics using artificial intelligence will enable precision hepatic medicine.

In 2012, Dutch scholar Lambin et al. first proposed the concept of Radiomics 25. Radiomics is a method that extracts large number of features from radiographic medical images using data- characterization algorithms 26, 27. These features, termed radiomic features, have the potential to uncover disease characteristics that failed to notice by naked eyes. The hypothesis of radiomics is that the distinctive imaging features between disease forms may be useful for predicting prognosis and therapeutic response for various conditions, thus providing valuable information for personalized therapy 28, 29. Radiomics method is used to analyze the results of preoperative CT examination in patients with liver tumors, and useful information was extracted and combined with clinical data to assist in the diagnosis of tumor properties. At the same time, a predictive model of lymph node metastasis of liver cancer can be established to achieve non-invasive and individualized prediction of lymph node metastasis as a predictive method for postoperative tumor recurrence and clinical prognosis. This approach helps to improve clinical decision making and provides valuable insights for subsequent individualized treatment and multi-disciplinary team (MDT) diagnosis and treatment (Fig. 1).

Pathology is the gold standard for tumor diagnosis. Due to the development of full-glass digital scanning system, data storage capacity has increased the advancement of graphic recognition technology, pathological slice recognition and evaluation based on artificial intelligence technology has a high accuracy 23, 24. Compared with traditional pathological diagnosis work, artificial intelligence has several advantages: (1) it can identify hidden feature textures and details that are currently not recognized by human eyes in pathological sections; (2) quantitatively described pathological features instead of qualitative grading; (3) The criteria are objective and consistent, avoiding geographical or subjective differences and/or biases. The identification and analysis of individual pathological sections by artificial intelligence technology may help to understand tumor heterogeneity and promote individualized medicine.

## 3D visualization and 3D printing in liver surgery applications

The 3D visualization technique of liver tumors refers to a tool for displaying, describing and interpreting the 3D anatomical and morphological features of liver tumors. Previous clinical imaging of liver tumors relied mainly on computer image processing technology such as B-ultrasound, CT and/or MRI, etc. This computer image data has been used to analyze and calculate data, and described the morphology and spatial distribution of targets such as liver, biliary tract, blood vessels and tumors 30. This method can separate the target of interest intuitively and precisely, and provide decision for accurate preoperative diagnosis, individualized surgical planning and surgical approach. However, surgeons can only rely on their experience to make an abstract of 3D based on their understanding of two-dimensional images. As the consequence of limitations and uncertainties of experience, it is difficult to evaluate complex liver tumors especially the diagnosis and preoperative planning, resulting in a relatively high incidence of postoperative complications. With the help of CT scanning technology, liver tumor scanning can now obtain higher resolution and larger images, and a large amount of diagnostic information using 3D technology. With this technology, diagnosis and treatment model of liver tumors has gradually shifted from the traditional two-dimensional to 3D visualization technology. In addition, 3D liver printing has enable a leap-forward transition from 3D visualization to 3D physical models, which can better improve the precision surgery of complex liver tumors 31.

In addition to obtaining basic diagnostic information such as CT and MRI, liver surgeons also need to recognize the local lesion itself and its adjacent organ details based on individual patient. Prior to the operation, detailed planning of the scope of the lesion and surgical procedure should be made to ensure a safe operation 30, 32. Currently, 3D visualization and 3D printing, as new digital medical technologies, play an increasingly important role in preoperative evaluation and surgical planning for liver surgery. After reconstruction by 3D visualization software, 3D printing of liver can restore the characteristics of organs in the body, making the human liver more realistic than 3D visualization. The advantages of this method are: (1) It can show the exact location, size and shape of the tumor and the relations between the tumor and vascular in all aspects. (2) It can provide intuitive navigation during surgery to quickly identify and locate key parts. Surgeons can use 3D visualization techniques to perform virtual simulations before surgery to accurately assess the resectability of the lesion and to select the lesion resection. 3D printing technology has shifted the transition from 3D images to 3D printed models. By comparing the 3D printed model with the intraoperative findings during hepatectomy, the surgeon can quickly identify, locate the lesion and determine the surgical resection plane. This provides a more intuitive real-time navigation for surgery, guiding the separation of important anatomical structures and removal of tumor lesions. This method can remove the lesion completely, avoid damage to important anatomical structures, improve surgical outcomes, and reduce the risk of surgery.

Erbay et al. found that intrahepatic vascular variability was as high as 70% 33. Due to the location of the lesion and the variation and displacement of adjacent vessels, the probability of intravascular vascular variability is even higher than intrahepatic vascular. Therefore, by applying a 3D reconstruction system of the liver before surgery, we can now display the full-scale stereoscopic information of the lesion. This can avoid accidental injury to the pipeline during surgery, reduce intraoperative bleeding, postoperative residual liver ischemia or necrosis, and is more conducive to accurate liver resection and shortened operation time. The author's team in 3D visualization technology for assisted surgical resection of complex liver cancer study has confirmed that the 3D reconstruction model can provide surgeons a stereoscopic, intuitive and accurate hilar anatomy, providing a visual solution for key surgical problems. Preoperative use of 3D visualization techniques to assess the portal vein, hepatic artery, bile duct type and variation, and the extent of longitudinal infiltration and lateral invasion of the tumor are important for the development of a high quality surgical plan. In addition, 3D visualization and virtual simulation surgery can provide new ideas for the treatment of liver cancer. Combined portal resection and reconstruction treatment is now widely applied in larger centers. However, combined hepatectomy and reconstruction treatment is still challenging due to its complications. Repeated simulation of surgical procedure and determination of the length of the revascularization are needed to improve the success rate and safety of the operation.

The degree of portal vein involvement and variation has a decisive influence on the choice of surgical approach for hilar cholangiocarcinoma. The portal vein separation limit point under normal conditions (U point: the left lateral branch of the portal vein and the sagittal corner; P point: right anterior branch of the portal vein, right posterior branch bifurcation) refers to the bile duct in the hepatectomy from the parallel portal vein and the extreme part of the hepatic artery that is peeled off. The bile duct beyond this limit point cannot be separated and must be cut off separately. The portal vein separation limit point under normal conditions refers to the extreme part of the bile duct that can be separated from the parallel portal vein and hepatic artery during hepatectomy (U point: the horizontal part of the left branch of the portal vein and the corner of the sagittal part; P point: the right front branch of the portal vein and the bifurcation of the right posterior branch). The bile duct beyond this limit cannot be separated and must cut off separately. In the case of portal vein variation, the portal vein separation limit point will move forward or downward. It is extremely important to identify the variation of the intravascular vascular structure to determine the exact location of the hepatic resection limit point. Based on the 3D reconstruction of portal vein, we propose to classify the adjacent relations between tumor and portal vein into 4 grades:

- 0 grade: tumor does not compress the portal vein;
- 1^st^ grade: tumor compression but does not invade the portal vein;
- 2^nd^ grade: tumor invades the portal vein trunk, but the continuity of the blood vessels is uninterrupted;
- 3^rd^ grade: tumor invades the portal vein trunk and interrupts the continuity of blood vessels.

The portal vein of patients with grade 0 or 1 has not been violated; patients with grade 2 or 3 require combined revascularization to achieve radical resection. The concept of vascular assessment centered in the portal vein is different from the concept of grading the portal vein and superior mesenteric vein proposed in laparoscopic pancreaticoduodenectomy. The latter is similar to the joint vascular assessment and exploration perspective, emphasizing the procedural and standardization of the surgical procedure. The portal vein is used as the axial center combined with the portal vein separation limit points (U point, P point) to evaluate the portal vein condition, emphasizing the development of individualized and radical surgery strategies for hilar cholangiocarcinoma 34.

### Application of indocyanine green molecular fluorescence in liver surgery

Optical imaging technology represented by near-infrared fluorescence imaging technology has been applied to liver surgery, providing technical support for real-time, high-precision surgical navigation. Indocyanine green (ICG) is a cyanine dye used in medical diagnostics. It is used to determine cardiac output, hepatic function, liver and gastric blood flow, and for ophthalmic angiography. These infrared frequencies penetrate retinal layers, allowing ICG angiography to image deeper patterns of circulation than fluorescein angiography. ICG binds tightly to plasma proteins and becomes confined to the vascular system. ICG has a half-life of 150 to 180 seconds and is removed from circulation exclusively by the liver in the form of bile juice.

ICG-mediated near-infrared light detection technology is widely used in surgical navigation 35, 36. After intravenous injection of ICG, ICG can be rapidly taken up by liver cells and finally excreted via the biliary system. Exogenous light with a wavelength range of 750-810 nm is used to excite the fluorescence during the operation, and the extrahepatic bile duct morphology is clearly displayed under fluorescence. If? one side of the bile duct is invaded by the tumor and the biliary excretion function is impaired, the ICG is targeted to stay in the liver tissue of the lesion side, and the delayed hemi fluorescence of the liver appears. In the radical operation of gallbladder carcinoma, the gallbladder neck often adheres to the common bile duct due to chronic inflammation. In order to safely and accurately dissect the bile duct tissue, molecular cholangiography can be used to clearly identify the common bile duct area and reduce bile duct injury. Compared with traditional cholangiopancreatography, choledochectomy with intravenous injection of ICG has prime advantages in shortening operation time and avoiding direct injection of contrast agent at bile duct to cause iatrogenic bile duct injury.

However, due to problems such as shallow penetration depth, tissue interference from fluorescent background, and poor imaging specificity, the application of fluorescent optical imaging in medicine is limited. However, a new technology was developed to overcome this limitation. Optical/ acoustic multimode technology, a system where it combines photoacoustic imaging technology with traditional ultrasound technology to enable high-precision imaging across molecules, cells, tissues, and organs. It can also quantitatively analyze the blood flow and oxygen metabolism of the lesion, and provide conditions for accurately determining the tumor boundary and guiding the accurate liver tumor resection.

### Virtual and Mixed Reality in hepatectomy

The anatomical structure of the hilar is complicated. Therefore, the 3D spatial relationship between the departmental vein, hepatic artery, hepatic vein and tumor in the liver of patients with biliary tract tumor is obtained and this information is the basis of surgical treatment for biliary malignant tumor. In liver surgery, augmented reality technology uses CT and MRI data to reconstruct a 3D image of the liver and intrahepatic vasculature for surgical planning, where the virtual image is superimposed in the surgical field for navigation. The 3D image of the surgical plan is superimposed on the liver surface in a 1:1 ratio, which can achieve the preliminary definition of the liver range of pre-excision of gallbladder cancer or hilar cholangiocarcinoma, and help the surgeon to achieve accurate liver resection. Laparoscopic surgery is the direction of biliary tumor surgery. For laparoscopic liver resection, in addition to tactile feedback, visual feedback is also critical for fine manipulation and vascular management. Augmented reality technology can project a 3D visualization liver model into the surgical area, which helps to solve the problem of uncoordinated hand and eye during surgery. The mixed reality presents the information of the virtual scene in the real environment, and establishes an information loop of interactive feedback between the real world, virtual world and user to enhance the realism of the user experience 32. The application of mixed reality in abdominal surgery is still in its early phase. This new technology displays a 3D model near the surgical site, reducing the shift between the operating space and visualization.

Virtual reality technology is a simulation system that can create and experience virtual worlds. It uses a virtual environment in which a computer simulates a real-world scene in three dimensions. It is a system simulation of multi-source information fusion, interactive 3D dynamic view and entity behavior that allows users to enter the environment. Virtual anatomy in virtual reality environments provides learners with a realistic 3D learning environment that allows surgeons to quickly master operational techniques and shorten training cycles.

3D visualization is the first step in creating virtual reality. By blending different images, surgeons can now develop strategies based on coherent, multimodal virtual views in conjunction to surgical conditions. The application of virtual reality, augmented reality and mixed reality provides a new surgical navigation method to reduce the uncertainty between 3D reconstruction model and actual operating space. It has great potential in preoperative planning, intraoperative navigation, surgical simulation training, and doctor-patient communication 32, 37.

### Predictive model guided postoperative management

Good postoperative management is also an important part of accurate liver resection. By analyzing the biological nature of the images, these imaging features are combined with clinical data to establish corresponding diagnostic and predictive models 38. This model can be used to determine tumor characteristics, postoperative complications, predict patient prognosis, etc., and strengthen perioperative management. Jeong et al 39. Used the Radiomics scoring system constructed by angiography to predict the risk of liver failure after hepatectomy and the risk of early recurrence after surgery, and came across intelligent diagnosis and treatment. This provides a new direction and ideas for the prevention of postoperative complications in liver tumor surgery.

### Wearable device guides rehabilitation after surgery

The postoperative health monitoring device can ensure to treat patients with vital signs abnormalities in a timely manner. Furthermore, wearable device can be used as effective tools to support postoperative rehabilitation training and rehabilitation evaluation 40. The emergence and development of wearable technology provides technical support for the realization of these needs. Wearable Devices refers to the integration of sensors, wireless communications, multimedia and other technologies into the daily wear of people's glasses, watches, bracelets, clothing and footwear, and can measure various signs. The wearable medical device can collect the physiological data of the human body through the sensor, and transmit the data wirelessly to the central processor. The central processor then sends the data to the medical center for a comprehensive, professional and timely analysis to determine further treatment plans. Blood glucose, pressure and oxygen level not only can be monitored using non-invasive continuous monitoring technology featured in wearable medical devices, but also with cloud storage technology to store and analyze data through the cloud. The wearable device can be connected to the hospital's medical record system and monitoring center, timely warnings, corresponding diagnosis and treatment opinions are provided if there is abnormality. For example, Rawe et al. used a pulsed radio frequency energy (PRFE) device to solve postoperative pain problems 41.

The wearable device can also develop a personalized follow-up timetable once the patient is discharged from the hospital and to guide patient for follow-up after surgery. This can help the medical field to establish a complete database of clinical pathology data and collect material for the construction of big data 15, 42.

## Discussion

The great advancement of biomedicine has given rise to evidence-based medicine and the return to patients' humanistic care; we see a profound change in diagnosis and treatment strategies, thinking modes and technical characteristics of traditional surgery, thus lays a new surgical paradigm. Surgery has shifted from the initial intuitive and empirical mode into the modern era. In the era of intelligent surgery, the surgeon's way of thinking will rise from image cognition and data cognition to new figurative cognition. With the continuous optimization of information computing capabilities, transmission speed and deep learning technology, the links between the various aspects of medical work will become closer and work efficiency will be significantly improved. Each medical institution is no longer an isolated closed system, but an open platform that is symbiotically born on the basis of big data sharing, enabling intelligent management of the entire data link of surgical practice. The transparency of information and neutralization of medical services will provide patients a better medical experience 42-44.

The digitization of surgery is a requirement for PM and it is a prerequisite for the development of surgery itself. Precision Liver Surgery is a multidisciplinary integrated system including information science and computer science. The emergence of a series of innovative intelligent diagnosis and treatment technologies has provided new strategies and means for disease diagnosis and treatment. As health is a priority, doctors are continually trying to find the ways to implement new technology and provide impactful result. The addition of digital medical technology has made hepatectomy more true to life. Digital intelligent diagnosis and treatment technology are used in the visualization of disease diagnosis, preoperative evaluation, surgical planning, real-time guidance of surgery, and training for young physicians. Deep learning gathers a massive volume of liver tumor data, including patient's laboratory data, image records, medical reports, outcomes and predicts complication. In addition, digitization allows patients to have better healthcare experience and a faster recovery.

**Figure 2:**
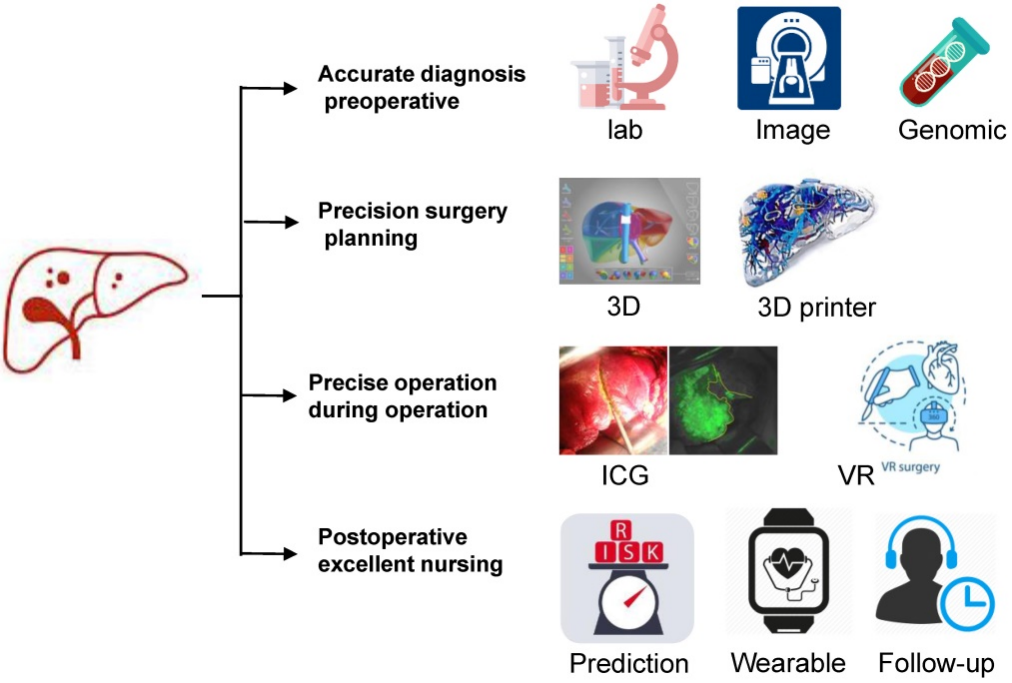
** Schematic diagram of precision hepatectomy.** As a new surgical concept and technical system, precision liver surgery aimed at providing accurate preoperative evaluation, precision surgery plan, fine surgical procedures and excellent postoperative management trying to pursue maximal effect/cost ratio and maximal liver-saving with minimal invasiveness.

Detailed evaluation of preoperative liver function and surgical plan, individualized operation, and postoperative intervention with molecular targeting are the key factors to achieve accurate surgical treatment of liver cancer (Fig. 2). We hope to integrate the unique genomic information and clinicopathological features of individual liver cancer patients to provide individualized surgery for each patient and truly achieve the precision of liver cancer treatment in the future.

## Abbreviations

PM: Precision medicine
DGE: Digital gene expression profiling
AI: Artificial intelligence
MDT: Multi-disciplinary team
ICG: Indocyanine green
CT: Computed Tomography
MRI: Magnetic Resonance Imaging
